# Effectiveness of Flat-Panel Fluoroscopy in Direct Anterior Total Hip Arthroplasty: A Comparison to Image Intensifier Fluoroscopy With Radiopaque Grid

**DOI:** 10.1016/j.artd.2023.101253

**Published:** 2023-11-06

**Authors:** Zachary A. Gapinski, Matthew S. Kerr, Joshua R. Langford, Frank R. Avilucea

**Affiliations:** Department of Orthopedic Surgery, Orlando Health Jewett Orthopedic Institute, Orlando, FL

**Keywords:** Total hip arthroplasty, Fluoroscopic grid, Flat panel, Component positioning

## Abstract

**Introduction:**

The use of traditional, image intensifier fluoroscopy with a radiopaque grid during direct anterior total hip arthroplasty (DA THA) has demonstrated reduced variability in component positioning and operative time compared to fluoroscopy without a grid. A disadvantage of image intensifier fluoroscopy is spatial distortion, particularly compared to flat-panel fluoroscopy systems. The purpose of this study is to determine whether flat-panel fluoroscopy decreases variability in component positioning during DA THA compared to the use of traditional grid fluoroscopy.

**Methods:**

We retrospectively reviewed 70 consecutive DA THAs between February 2020 and February 2021: 36 using flat-panel fluoroscopy, and 34 using traditional fluoroscopy with a grid. Radiographs were independently reviewed by 2 authors to identify components exceeding goal parameters: cup abduction of 40 ± 10 degrees, as well as offset and limb lengths within 10 mm of the contralateral side. Binary values for goal parameter achievement were assigned for each THA.

**Results:**

No significant difference was observed in the number of hips that met goals for cup abduction (100% vs 97%, *P* = 1.00), hip offset (88% vs 88%, *P* = 1.00), limb length (91% vs 94% [ ±10 mm], *P* = .669, 65% vs 72% [±5 mm], *P* = .498), or for the number of hips that met all 3 component goals (79% vs 80%, *P* = 1.00). No significant difference in operative time was noted between the 2 groups (110.2 minutes vs 100.9, *P* = .76).

**Conclusions:**

We demonstrated no significant difference in component positioning in DA THAs utilizing flat-panel fluoroscopy as compared to using traditional fluoroscopy with a grid.

## Introduction

Rates of total hip arthroplasty (THA) are anticipated to rise over the coming years, with an expected 635,000 procedures being performed in 2030 [1]. Hip offset, limb length equality, and acetabular component positioning are of critical importance for THA clinical outcomes and implant longevity [2,3]. Correct component positioning can be achieved in several ways. Templating and preoperative cross-sectional imaging have been shown to reduce variability in component position [[4], [5], [6]]. In addition, surgeons use a variety of intraoperative methods to achieve improved component positioning including the use of anatomical landmarks, intraoperative mechanical devices, computed tomography–based navigation, robotics, and the use of intraoperative fluoroscopy [4,[7], [8], [9]].

**Figure 1:**
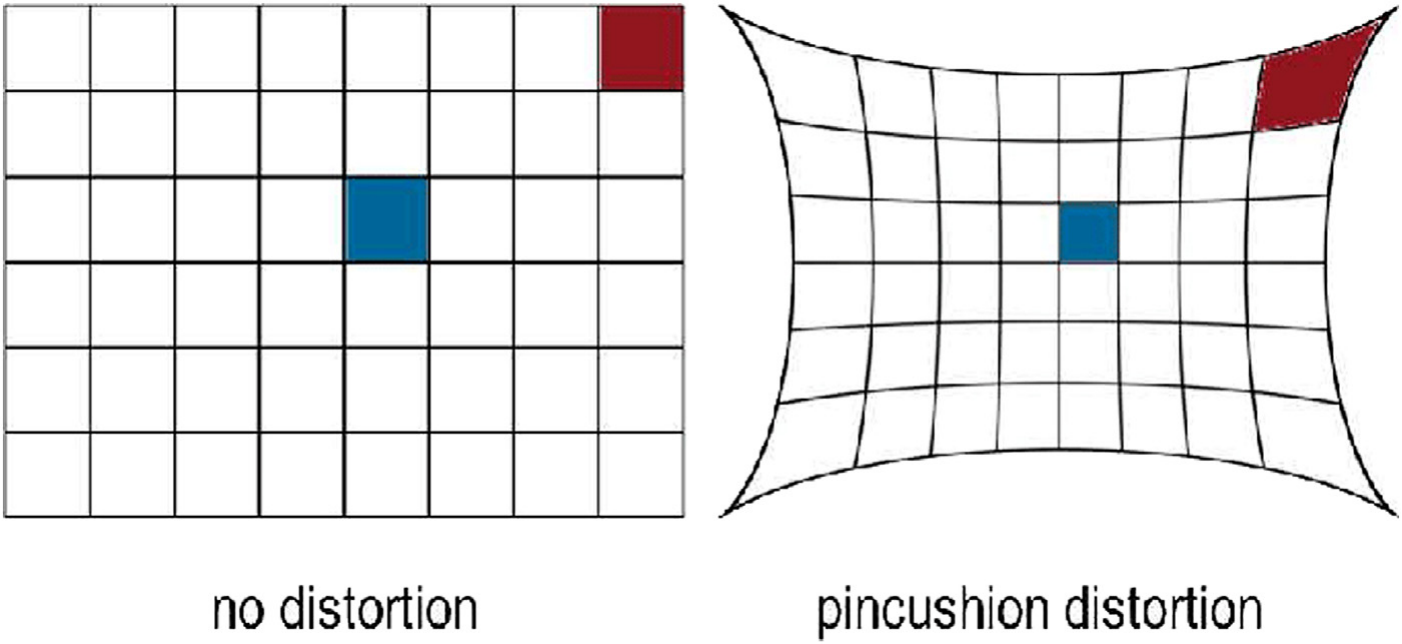
Pin cushion distortion created by a large-field image intensifier system (right) vs distortion-free image created by a large-field flat-panel fluoroscopic system (left).

**Figure 2:**
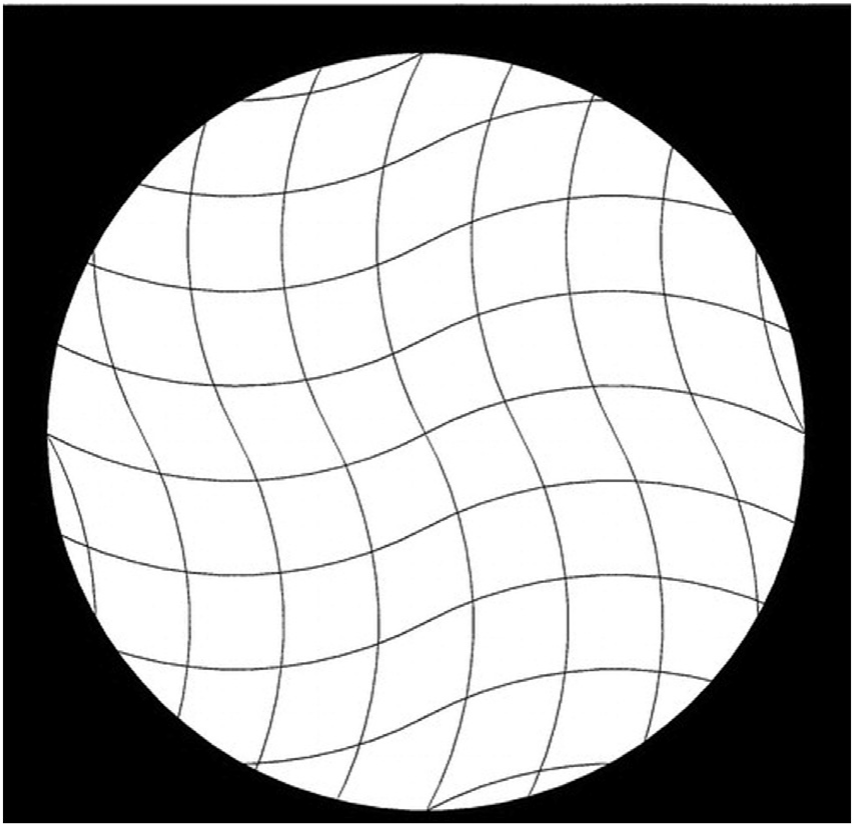
S-distortion in image intensifier systems caused by extraneous magnetic fields.

Specifically, the use of traditional, image intensifier fluoroscopy with a radiopaque grid is a common system utilized by surgeons performing supine-positioned direct anterior THA (DA) and has been shown to reduce variability in component positioning [9]. Unfortunately, image intensifier radiographic systems are susceptible to numerous image distortions. Pincushion distortion, which creates geometric distortion at the periphery of the image, is created by the mapping of electrons from a concave photocathode to a planar output screen (Fig. 1). Image intensifier systems are also subject to a second form of distortion called S-distortion caused by extraneous magnetic fields (Fig. 2) [10]. A grid attachment can be placed on these image intensifier machines to mitigate the effect of distortions on THA component positioning.

Flat-panel detectors are a newer technology that eliminate spatial distortion and provide an image of higher resolution [11]. The use of flat-panel fluoroscopy has become increasingly prevalent in orthopedics; however, no studies have examined its use in supine THA. The purpose of this study is to determine whether flat-panel fluoroscopy decreases variability in component positioning during direct anterior THA compared to the use of image intensifier fluoroscopy with a radiopaque grid.

## Material and methods

We retrospectively reviewed 118 consecutive DA THAs between February 2020 and February 2021. Each surgery was performed by one of 2 surgeons (J.R.L. and F.R.A.) using the direct anterior approach to the hip on a Hana table (Mizuho OSI, Union City, CA). The same preoperative and postoperative protocols were implemented for each study arm. All THAs were performed using cementless femoral and acetabular components. Flat-panel or traditional fluoroscopy with grid was chosen based on surgeon preference.

Patients were identified using the Current Procedural Terminology (CPT) code 27130, arthroplasty, acetabular, and proximal femoral prosthetic replacement (THA), with or without autograft or allograft, and the CPT code 27132 conversion of previous hip surgery to THA, with or without autograft or allograft.

Patients included in the study were aged 18 years or older and underwent THA via the direct anterior approach. Exclusion criteria included surgical approach other than direct anterior, revision hip arthroplasty or patients who did not have adequate anteroposterior (AP) pelvis radiographs at follow-up. Of the 118 patients, 48 were excluded due to the lack of follow-up or absence of appropriate postoperative radiographs.

Intraoperative fluoroscopic images were obtained in a similar manner by each surgeon. An AP pelvis radiograph was obtained prior to prepping and draping the operative hip to ensure the pelvis was not mal-rotated and to compare to preoperative templates. Fluoroscopic images were utilized during acetabular reaming and during acetabular component placement to ensure appropriate positioning. Fluoroscopic images were also obtained during trialing of femoral components and following final implant placement and reduction of the THA. Leg length discrepancy was determined using the trans-pelvic line, and THA offset was estimated based off contralateral hip offset intraoperatively. Traditional fluoroscopy was obtained using a GE OEC 9900 C-arm (General Electric Healthcare, Waukesha, WI) with a grid accessory (OrthoGrid, Salt Lake City, UT). Flat-panel fluoroscopy was obtained using a Ziehm Vision RFD C-arm (Nurnberg, Germany)

Postoperative radiographs were obtained immediately following surgery and at each follow-up appointment. Acceptable AP radiographs of the pelvis were those with legs positioned at 15 degrees of internal rotation and with the coccyx centered within 2 cm of the symphysis pubis. Radiographs were independently reviewed by 2 authors to measure several factors associated with implant position and restoration of alignment: cup abduction, femoral offset, and limb length defined by the position of the lesser trochanteric profiles.

**Figure 3:**
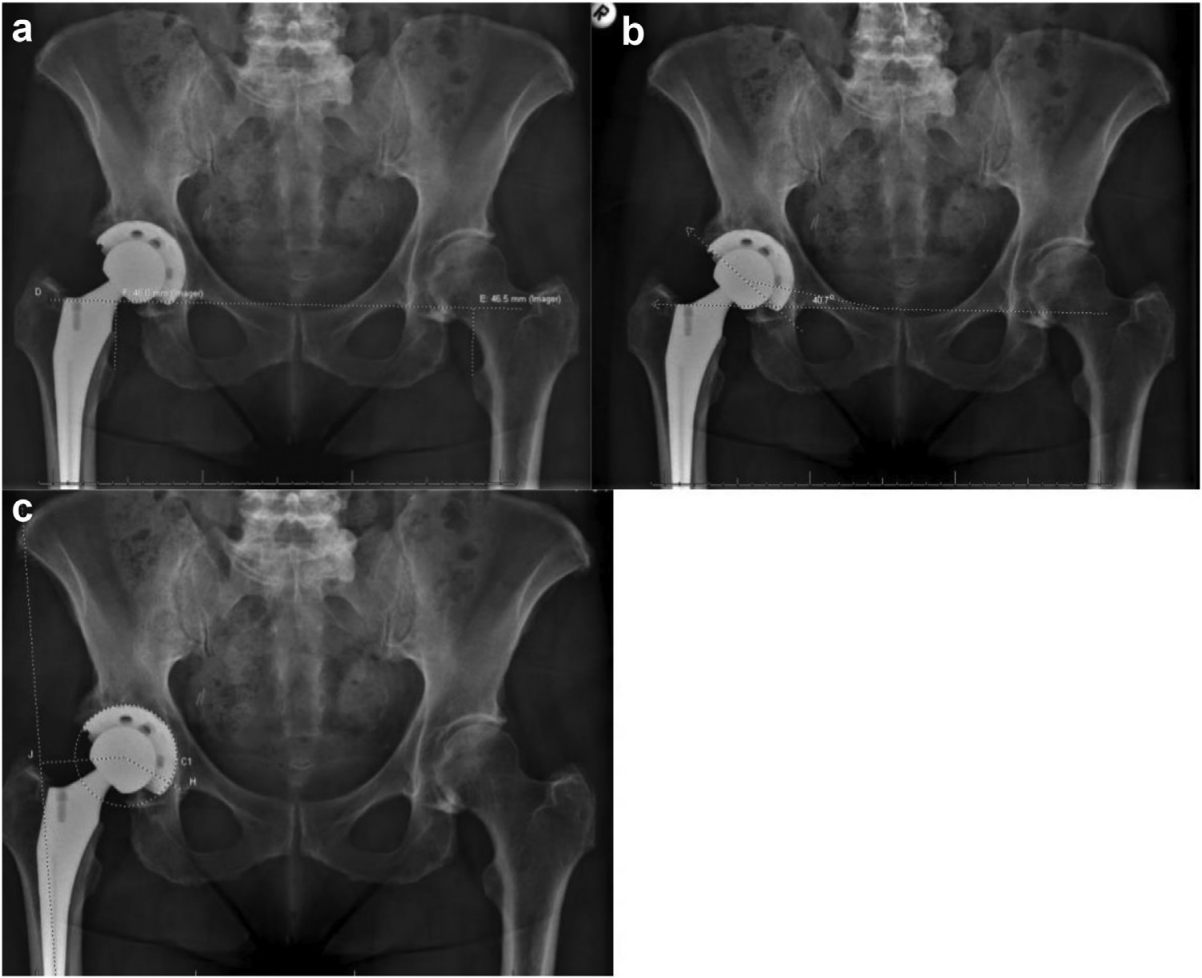
Post operative radiographs showing measurements of limb length (A), cup abduction (B), and hip offset difference (C).

Two independent readers (Z.A.G. and M.S.K.) blinded to each patient’s grouping measured cup abduction, limb length, and hip offset difference compared to the contralateral limb. The measurement method was similar to that used by Gilliland et al. [9]. Limb length was measured as the perpendicular distance in the teardrop line to the apex of the lesser trochanter. Hip offset was measured by the femoral offset added to the horizontal position of the center of rotation (COR) of the hip (Fig. 3). Hip offset was measured in lieu of femoral offset because femoral offset does not represent the true displacement of the femur from the pelvis because this offset is influenced by both femoral offset and COR [12]. The position of the COR was measured as a distance parallel to the teardrop line from the teardrop to the center of the acetabulum component.

The goal for cup abduction angle was 40 degrees ± 10 degrees based on a standard set by Lewinnek et al. [13]. The goal of limb length and hip offset was to be within 10 mm of the contralateral side. A secondary limb length goal of 5 mm was set to further distinguish between the groups. Binary values within or out of goal parameters were assigned for each THA predicated on whether the measurements were within the range of acceptability. Ideally, a THA would be within all 3 range goals.

Along with obtaining demographic information, records were reviewed for operative start and end time, allowing calculation of surgical time for each case.

An independent statistician analyzed the data utilizing a commercially available software program (SPSS; IBM Inc., Armonk, NY). Student *t* test was used for comparing continuous variables such as heigh, weight, Body Mass Index, operative time, and age. Fisher exact test was used to compare binary values. No multivariate analysis was performed as there were no significant differences found between the 2 groups.

## Results

After exclusions, 70 patients—36 using flat-panel fluoroscopy and 34 using traditional fluoroscopy with a grid—were included in the study. There were no demographic differences between these 2 groups including age, Body Mass Index, gender, or etiology of arthritis (Table 1).

**Table 1:** Demographics/diagnosis means and standard deviations/percentages and cell sizes provided.

Demographics	Grid (*n* = 36)	Flat panel (*n* = 34)	*P* value
Age, mean (SD)	62.8 (10.9)	61.9 (9.9)	.729
Gender, % (*n*)			
Female	61.1% (22)	50.0% (17)	.350
Male	38.9% (14)	50.0% (17)	
Body Mass Index (BMI), mean (SD)			
Height (cm)	168.6 (10.4)	168.8 (12.7)	.923
Weight (kg)	94.4 (20.7)	90.5 (19.7)	.414
BMI	33.5 (8.3)	32.0 (8.2)	.139
Diagnosis, % (*n*)			
Osteoarthritis	91.7% (33)	79.4% (27)	.219
Fracture	8.3% (3)	8.8% (3)	
Avascular necrosis	0.0% (0)	2.9% (1)	
Post-traumatic	0.0% (0)	8.8% (3)	

No significant difference was seen between flat-panel and grid groups for number of hips that met the goal for cup abduction of 30-50 degrees (100% vs 97%, *P* = 1.00). Hip offset restoration to within 10 mm of contralateral extremity was found to have no statistically significant difference (88% vs 88%, *P* = 1.00). Restoration of limb length to 10 mm of the contralateral extremity had no statistically significant difference either (91% vs 94%, *P* = .669). Restoration of limb length to 5 mm of the contralateral side also showed no significant difference (72% vs 65%, *P* = .498). Lastly, the number of hips that met all 3 component goals showed no difference between groups when considering limb length ± 10 mm (79% vs 80%, *P* = 1.00) (Table 2). The number of hips meeting all 3 component goals with a limb length goal of 5 mm also showed no difference (64% vs 53%, *P* = .353).

**Table 2:** Goals means and standard deviations/percentages and cell sizes provided.

Parameter	Grid (*n* = 36)	Flat panel (*n* = 34)	*P* value
Cup abduction (30-50), % (N)	97% (35)	100% (34)	1.000
Limb length (LL) % ± 10 mm (N)	94% (34)	91% (31)	.669
LL % ± 5 mm (N)	72% (26)	65% (22)	.498
Hip offset goal % (N)	88% (32)	88% (30)	1.000
Within all 3 goals % LL ± 10 mm (N)	80% (29)	79% (27)	1.000
Within all 3 goals % LL ± 5 mm (N)	64% (23)	53% (18)	.353

No significant difference in operative time was noted between flat-panel vs grid groups (110.2 minutes vs 100.9, *P* = .76) (Table 3).

**Table 3:** Operative times, mean and standard deviation provided.

	Grid (*n* = 36)	Flat panel (*n* = 34)	*P* value
Operative time (min) Mean (SD)	100.9 (21.0)	110.2 (22.3)	.076

## Discussion

Hip offset, limb length equality, and acetabular component positioning are of critical importance for THA outcomes, implant longevity, and patient satisfaction. Fluoroscopy is a commonly used tool to examine these factors. Matta et al. described the overlay method utilizing fluoroscopy, followed by printing an AP image of trial components, and superimposing the image over a contralateral hip to compare length and offset [8]. This technique has been reported to enable matching of length and offset to the native hip [8]. The difficulty with the technique is the need to print AP images of each hip, create the overlay, and measure differences to identify changes needed for the reconstruction.

Gililland et al. aimed to make this process more efficient by using an intraoperative fluoroscopic grid to assess cup abduction, limb length, and hip offset [9]. The group reported the use of the grid significantly reduced component position variability regarding cup abduction, limb length, and hip offset. Moreover, compared to the use of fluoroscopy alone, target acetabular cup position was achieved in 97% vs 83% of cases. Our study corroborates the effectiveness of grid as we were able to achieve ideal cup position in 97% of cases. The lone use of the flat-panel technology, however, also enabled the surgeon to also achieve such a cup position in 100% of cases.

In terms of limb length, Gililland et al. found 100% restoration of limb length within 10 mm of contralateral extremity in the grid group vs 88% with fluoroscopy alone [9]. Our study identified similar results but no statistical significance between the 2 groups with 91% and 94% limb length restoration within 10 mm of contralateral extremity in flat panel and grid, respectively. To further distinguish between groups, a secondary goal of limb length restoration to 5 mm within the contralateral extremity was set. Similar restoration was achieved in both groups with 65% in the flat-panel group and 72% in the traditional fluoroscopy with grid group.

Hip offset restoration to within 10 mm of the contralateral extremity was also significantly higher in the grid group for Gilliland with 85% vs 67% with the fluoroscopy group. We were able to achieve 88% hip offset restoration to within 10 mm of contralateral extremity in both groups. Gilliland identified statistical significance in operative time between the grid and fluoroscopy groups. In our study, we did not observe any difference in operative time between groups, an expected finding as neither group required extra steps to be taken intraoperatively.

In a survey of modern fluoroscopy, Nickoloff details physics of flat-panel fluoroscopy systems and denotes differences from imaging intensifier systems [11]. Flat-panel systems have multiple advantages including smaller size, extended dynamic range, no spatial distortion, and greater stability [11]. In fact, the spatial distortion created by image intensifier systems makes it difficult to reliably compare sides to achieve correct implant position and alignment. This distortion necessitates the use of grid accessories that can be added to machines or operative tables for improved component positioning. Our study showed that flat-panel fluoroscopy is equally effective to image intensifier systems with a grid.

There were several limitations to this study. The study was retrospective in nature. We had 48 patients excluded from the study due to the lack of follow-up or missing postoperative radiographs. Although our radiograph technicians follow a standardized technique when obtaining AP pelvis radiographs, subtle differences in rotation of the pelvis may have affected the measurements. It should also be noted that the grid utilized by Gilliland was affixed to the operative table, whereas the grid utilized in our study was affixed to the fluoroscopy machine. To date, no studies have been performed comparing how such positioning differences would affect the different types of fluoroscopic grids utilized in DA THA.

## Conclusions

In conclusion, we demonstrated equally favorable surgical outcomes regarding acetabular component position, restoration of limb length, and hip offset and no added operative time in DA THAs utilizing flat-panel fluoroscopy as compared to using traditional fluoroscopy with a grid. We recommend surgeon preference in determining intraoperative fluoroscopic technique.

## Conflicts of interest

The authors declare there are no conflicts of interest.

For full disclosure statements refer to https://doi.org/10.1016/j.artd.2023.101253.

## Appendix A Supplementary data

Conflict of Interest Statement for Kerr

Conflict of Interest Statement for Gapinski

Conflict of Interest Statement for the Authors Langford and Avilucea
