# Supplementary material for: Effectiveness of Flat-Panel Fluoroscopy in Direct Anterior Total Hip Arthroplasty: A Comparison to Image Intensifier Fluoroscopy With Radiopaque Grid
Source: Arthroplast Today. 2023 Nov 6;24:101253. doi: 10.1016/j.artd.2023.101253 (PMC10665650; doi:10.1016/j.artd.2023.101253)
Supplement: Conflict of Interest Statement for Kerr [file mmc1.pdf]

INDIVIDUAL CONFLICT OF INTEREST STATEMENT

American Association of Hip and Knee Surgeons

(Adopted from the American Academy of Orthopaedic Surgeons disclosure statement)

The following form **must be filled out completely and submitted by each author (example, 6 authors, 6 forms). All items require a response. If there is no relevant disclosure for a given item, enter "*None.*"**

Manuscript Title

1. Royalties from a company or supplier (The following conflicts were disclosed)

    None

2. Speakers bureau/paid presentations for a company or supplier (The following conflicts were disclosed)

    None

3A. Paid employee for a company or supplier (The following conflicts were disclosed)

    None

3B. Paid consultant for a company or supplier (The following conflicts were disclosed)

    None

3C. Unpaid consultants for a company or supplier (The following conflicts were disclosed)

    None

4. Stock or stock options in a company or supplier (The following conflicts were disclosed)

    None

5. Research support from a company or supplier as a Principal Investigator (The following conflicts were disclosed)

    None

6. Other financial or material support from a company or supplier (The following conflicts were disclosed)

    None

7. Royalties, financial or material support from publishers (The following conflicts were disclosed)

    None

8. Medical/Orthopaedic publications editorial/governing board (The following conflicts were disclosed)

    None

9. Board member/committee appointments for a society (The following conflicts were disclosed)

    None

Each author must sign AND print or type his/her name, date and submit a separate form

In addition, one BLINDED Conflict of Interest form (no author names used) should be submitted per manuscript with all author disclosures.

**Matthew Kerr**	[signature]	**4/10/23**
Author Name (Print or Type)	Author Signature	Date
